# A Projective-Geometry-Aware Network for 3D Vertebra Localization in Calibrated Biplanar X-Ray Images

**DOI:** 10.3390/s25041123

**Published:** 2025-02-13

**Authors:** Kangqing Ye, Wenyuan Sun, Rong Tao, Guoyan Zheng

**Affiliations:** Institute of Medical Robotics, School of Biomedical Engineering, Shanghai Jiao Tong University, Shanghai 200240, China; yekangq@sjtu.edu.cn (K.Y.); wenyuansun1998@sjtu.edu.cn (W.S.); rongt@nvidia.com (R.T.)

**Keywords:** landmark localization, biplanar X-ray imaging, projective geometry

## Abstract

Current Deep Learning (DL)-based methods for vertebra localization in biplanar X-ray images mainly focus on two-dimensional (2D) information and neglect the projective geometry, limiting the accuracy of 3D navigation in X-ray-guided spine surgery. A 3D vertebra localization method from calibrated biplanar X-ray images is highly desired to address the problem. In this study, a projective-geometry-aware network for localizing 3D vertebrae in calibrated biplanar X-ray images, referred to as ProVLNet, is proposed. The network design of ProVLNet features three components: a Siamese 2D feature extractor to extract local appearance features from the biplanar X-ray images, a spatial alignment fusion module to incorporate the projective geometry in fusing the extracted 2D features in 3D space, and a 3D landmark regression module to regress the 3D coordinates of the vertebrae from the 3D fused features. Evaluated on two typical and challenging datasets acquired from the lumbar and the thoracic spine, ProVLNet achieved an identification rate of 99.53% and 98.98% and a point-to-point error of 0.64 mm and 1.38 mm, demonstrating superior performance of our proposed approach over the state-of-the-art (SOTA) methods.

## 1. Introduction

Biplanar X-ray imaging is widely used in image-guided spine surgery due to its low radiation dose and acquisition cost [1]. However, the lack of 3D information negatively affects navigation accuracy [2], which can be addressed by localizing 3D anatomical landmarks like vertebral body centers. The localization of landmarks in 3D space facilitates 2D/3D registration [3], 3D reconstruction [4,5,6,7,8,9], surgical navigation [2], and spinal geometry estimation [10].

Various Deep Learning (DL)-based methods have been developed for vertebra localization in both single [11,12,13,14,15,16] and biplanar [4,17,18,19,20,21,22] X-ray images. Payer et al. [13] proposed the SpatialConfiguration-Net (SCN) for medical landmark localization in a single image, which achieved superior performance and inspired various vertebra localization methods [14,15]. Unlike single-view localization methods, an effective biplanar fusion module is required in dual-view localization methods. The approaches to localizing landmarks in 3D space from calibrated biplanar images is generally divided into two categories: coordinate-level fusion methods and feature-level fusion methods. The coordinate-level fusion methods [5,6,17,18] triangulate coordinates of landmarks detected by a 2D single-view localization method, while the feature-level fusion methods [4,19,20,21,22] integrate features from both images, facilitating aggregation of biplanar information in the feature space. Given their advantages, this paper primarily focuses on the feature-level fusion methods.

Feature-level fusion is commonly achieved by either concatenating 2D features from each view [4,19,20,21,22,23,24] or constraining the landmark prediction using a consistency condition based on the assumption of orthogonality [25]. By concatenating images of both views, Aubert et al. [4,19] and Galbusera et al. [24] utilized biplanar information in vertebra localization. Furthermore, Wu et al. [20] proposed the X-module, which combined biplanar feature integration through summation and concatenation, thereby enhancing adolescent scoliosis assessments. The X-module has been adopted in other studies as well [21,22]. Huang et al. [25] achieved biplanar fusion in intraoperative long-film X-ray images by ensuring identical z-coordinates for the vertebrae in a Faster R-CNN framework. Despite these efforts, current methods neglect the projective geometry between views, failing to align the features from both views. Recently, a few multi-view fusion methods [26,27] have been developed in multi-view 3D human pose estimation. However, directly applying them to the biplanar X-ray localization task may lead to suboptimal results due to the non-informative features [26] and the lack of 3D information [27]. Table 1 provides a comparative overview of the state-of-the-art (SOTA) dual-view localization methods, in terms of their backbone networks, fusion level, and fusion strategies.

In this paper, an end-to-end projective-geometry-aware network, referred to as ProVLNet, is proposed for 3D vertebra localization in calibrated biplanar X-ray images. The design of ProVLNet features three components: a Siamese 2D feature extractor, a spatial alignment fusion module, and a 3D landmark regression module. The workflow of the proposed method begins with extracting features from both anterior–posterior (AP) and lateral (LAT) images through two weight-sharing 2D feature extractors. The output features are then unprojected and fused into 3D aggregated features by the spatial alignment fusion module. Finally, the 3D landmark regression module computes 3D coordinates from these aggregated features.

Our contribution can be summarized as follows:

- A novel end-to-end network called ProVLNet is proposed, which incorporates projective geometry to localize vertebrae in 3D space from calibrated biplanar X-ray images.
- A spatial alignment fusion module and a 3D landmark regression module are carefully designed, aiming to capture underlying 3D information by aligning 2D features from biplanar views in 3D space and to resolve semantic ambiguity in 3D landmark detections.
- Comprehensive experiments were conducted on two typical yet challenging datasets acquired from the lumber and the thoracic spine, demonstrating superior performance of our proposed approach over the state-of-the-art (SOTA) methods.

## 2. Method

### 2.1. Architecture Overview

**Figure 1:**
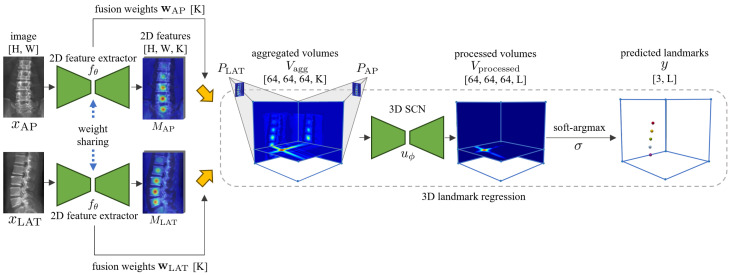
A schematic illustration of the overall pipeline of the proposed ProVLNet. The yellow arrows represent the spatial alignment fusion module. SCN represents the SpatialConfiguration-Net. Dimensions of data are indicated within square brackets.

Figure 1 presents the overall pipeline of ProVLNet. Although the network can be easily extended to multi-view scenarios, this paper mainly focuses on the widely used biplanar setup. The inputs to ProVLNet are anterior–posterior (AP) and lateral (LAT) X-ray images $x_{i}\in R^{H\times W},i\in \left\{\mathrm{AP},\mathrm{LAT}\right\}$ (*H* and *W* represent the spatial dimension) and the associated projection matrix $P_{i}\in R^{3\times 4}$ which can be used to project 3D coordinates to 2D image space. The overall pipeline of our method is as follows. First, two weight-sharing 2D feature extractors generate 2D features $M_{i}=f_{\theta }\left(x_{i}\right)\in R^{H\times W\times K}$ and the associated fusion weights $w_{i}\in R^{K}$, where K is the number of feature channels. Then, a spatial alignment fusion module unprojects these 2D features into 3D space to generate the corresponding 3D features $V_{i}\in R^{64\times 64\times 64\times K}$ based on projective geometry. From the unprojected 3D features $V_{i}$ and the associated fusion weights $w_{i}$, a weighted summation is calculated to obtain the fused 3D features $V_{\mathrm{agg}}\in R^{64\times 64\times 64\times K}$. Finally, a 3D landmark regression module, which includes a 3D SCN $u_{\phi }$ [13] with parameters ϕ and a soft-argmax function σ, is used to regress the 3D coordinates of all L landmarks. In particular, the 3D SCN regresses distinct heatmaps $V_{\mathrm{processed}}\in R^{64\times 64\times 64\times L}$ from the 3D fused features $V_{\mathrm{agg}}$, which are taken as the input to the soft-argmax function to calculate the landmark coordinates $y\in R^{3\times L}$. Our network is fully differentiable and supports end-to-end training, which can be formulated as:

(1) $$y=\sigma \left(u_{\phi }\left(F_{w}\left(f_{\theta }\left(x_{\mathrm{AP}}\right),P_{\mathrm{AP}},f_{\theta }\left(x_{\mathrm{LAT}}\right),P_{\mathrm{LAT}}\right)\right)\right),$$

where F(·) represents the spatial alignment fusion module.

### 2.2. 2D Feature Extractor

A Siamese-architecture-based 2D feature extractor is designed to extract features from the AP and the LAT images. Features extracted by the two weight-sharing 2D feature extractors are fed into the spatial alignment fusion module as described in the next section.

**Figure 2:**
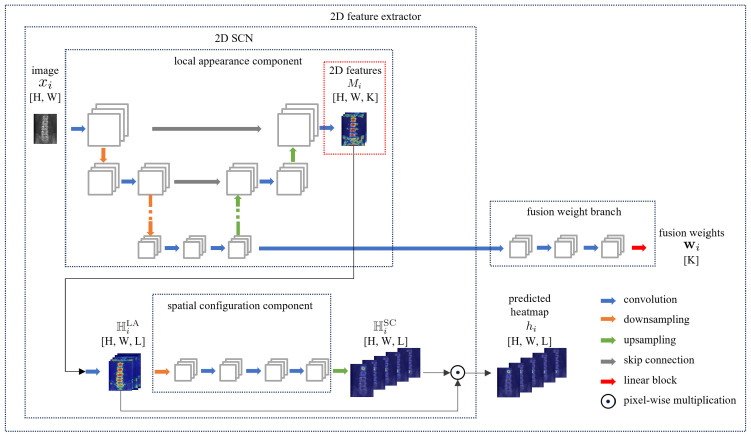
A schematic illustration of the network architecture of the 2D feature extractor, which takes a 2D SCN as the backbone. $H_{i}^{\mathrm{LA}}$ represents the output heatmap of the local appearance component, and $H_{i}^{\mathrm{SC}}$ represents the output heatmap of the spatial configuration component. The architecture of the local appearance component of the 2D SCN is a 5-layer U-Net [29]. The spatial configuration component includes an average pooling layer that downsamples the features, three convolutional layers, and an upsampling layer that rescales the features to their original size. Empty boxes represent intermediate features. Dimensions of data are indicated within square brackets.

Figure 2 illustrates the network architecture of our 2D feature extractor, which takes a 2D SCN as the backbone to produce local appearance features. In the 2D SCN, the heatmap produced by the local appearance component $H_{i}^{\mathrm{LA}}\in R^{H\times W\times L}$ is multiplied with the heatmap produced by the spatial configuration component $H_{i}^{\mathrm{SC}}\in R^{H\times W\times L}$ to generate the predicted heatmap $h_{i}\in R^{H\times W\times L}$. Moreover, the 2D features of the last layer before $H_{i}^{\mathrm{LA}}$ are denoted as $M_{i}\in R^{H\times W\times K}$, which are unprojected into 3D space to obtain the 3D features $V_{i}$ as described below. Additionally, a fusion weight branch is designed to generate $w_{i}\in R^{K}$ from the bottom-level features of the local appearance component. Both $V_{i}$ and $w_{i}$ are taken as the input to the spatial alignment fusion module to calculate a weighted 3D feature aggregation.

### 2.3. Spatial Alignment Fusion Module

**Figure 3:**
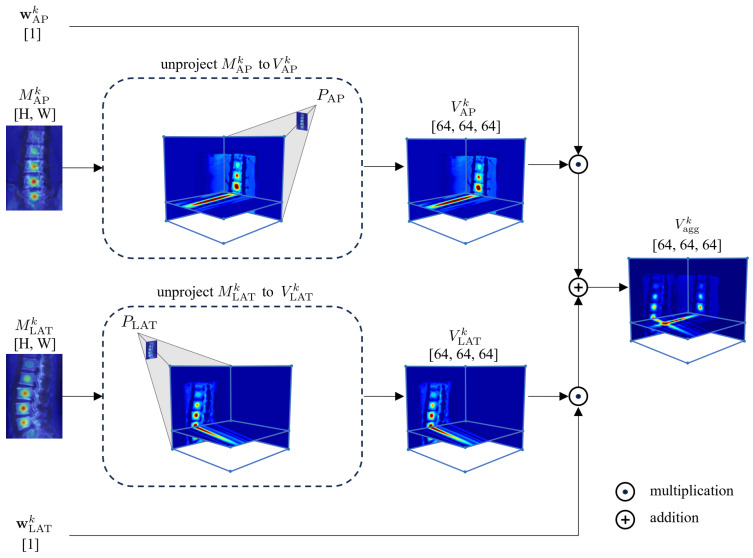
The aggregation of the 2D features $M_{i}^{k}$ in the spatial alignment fusion module. Dimensions of data are indicated within square brackets.

In this module, 2D features $M_{i}\in R^{H\times W\times K},i\in \left\{\mathrm{AP},\mathrm{LAT}\right\}$ extracted from the AP and the LAT images are unprojected into 3D features $V_{i}\in R^{64\times 64\times 64\times K}$ to incorporate projective geometry. These 3D features are then fused to produce $V^{\mathrm{agg}}\in R^{64\times 64\times 64\times K}$. An example of the *k*-th (k∈[1,K]) channel of $V^{\mathrm{agg}}$ is shown in Figure 3. Initially, a cubical volume is defined in 3D space as the target for feature unprojection. The center of the cube is determined using a linear algebraic triangulation approach [26] based on the centers of the AP and the LAT images. The 64×64×64 voxel cube represents a physical space of 250×250×250 ${\mathrm{mm}}^{3}$, capable of containing all vertebrae imaged by biplanar X-ray images in our experimental setup.

To unproject the *k*-th channel of 2D features $M_{i}$, denoted as $M_{i}^{k}$, into the *k*-th channel of the 3D features $V_{i}$, denoted as $V_{i}^{k}$, correspondences between voxels in $V_{i}^{k}$ and pixels in $M_{i}^{k}$ are established as follows. Specifically, by applying the defined center, physical dimensions, and size of the cube, the 3D coordinate $r_{v}^{3D}\in R^{3}$ of voxel *v* in $V_{i}^{k}$ is obtained. Then, the projection matrix $P_{i}$ is utilized to project the 3D coordinate $r_{v}^{3D}$ into the 2D coordinate $r_{v}^{2D}\in R^{2}$. The value of voxel *v* is set to the value of the pixel at $r_{v}^{2D}$ in $M_{i}^{k}$, which is obtained through bilinear sampling.

To account for the influence of different features across two distinct views, a weighted summation of $V_{i}^{k}$ is computed to obtain the 3D aggregated feature $V_{\mathrm{agg}}^{k}\in R^{64\times 64\times 64}$, with weights $w_{i}^{k}$ learned from the 2D feature extraction process (Figure 3):

(2) $$V_{\mathrm{agg}}^{k}=\sum_{i}\left(w_{i}^{k}\cdot V_{i}^{k}\right)/\sum_{i}w_{i}^{k}.$$

### 2.4. 3D Landmark Regression

In order to accurately localize landmarks from the 3D aggregated feature $V_{\mathrm{agg}}^{k}$, one has to reduce the ambiguity by suppressing false positive responses in the areas with similar structures. This is achieved by employing a 3D SCN, which has a two-branch structure that is well suited for this task, to process $V_{\mathrm{agg}}$ with the ultimate goal to produce 3D heatmaps $V_{\mathrm{processed}}\in R^{64\times 64\times 64\times L}$ for L landmarks.

The structure of the 3D SCN is similar to the 2D SCN used in the 2D feature extractor, where the convolutional layers in the 2D SCN are replaced by their 3D counterparts. With a large receptive field, the spatial configuration component of the 3D SCN robustly predicts the coordinate of a single landmark out of all landmarks in $H^{\mathrm{LA}}$. Such a design naturally incorporates the underlying 3D information.

In order to maintain the differentiability of the entire network, a soft-argmax function is employed instead of argmax to extract landmark coordinates from $V_{\mathrm{processed}}$. The first step is to compute the softmax across the spatial axes:

(3) $$V_{\mathrm{processed}}^{\prime l}=\exp\left(V_{\mathrm{processed}}^{l}\right)/\left(\sum_{64}\sum_{64}\sum_{64}\exp\left(V_{\mathrm{processed}}^{l}\right)\right),$$

where *l* represents the *l*-th (l∈[1,L]) channel.

Then, the centroid of $V_{\mathrm{processed}}^{\prime l}$ is calculated to obtain the predicted landmark $y^{l}\in R^{3}$, which is approximately the argmax point:

(4) $$y^{l}=\sum_{64}\sum_{64}\sum_{64}r\cdot V_{\mathrm{processed}}^{\prime l}\left(r\right),$$

where $r={\left(r_{x},r_{y},r_{z}\right)}^{T}$ represents the world coordinate of the voxel in volumes.

### 2.5. Loss

The total loss is the aggregation of the losses from the Siamese 2D feature extractor and the 3D landmark regression:

(5) $$L_{\mathrm{total}}=L_{2D}+L_{3D}.$$

The 2D loss $L_{2D}$ measures the similarity between the SCN’s predicted heatmaps $h_{i}\in R^{H\times W\times L}$ and the ground truth Gaussian heatmaps $g_{i}\in R^{H\times W\times L}$. To resolve the foreground–background class imbalance, a combination of Dice loss and Mean Squared Error (MSE) loss is used:

(6) $$L_{2D}=\sum_{i}L_{\mathrm{Dice}}\left(h_{i},g_{i}\right)+\sum_{i}L_{\mathrm{MSE}}\left(h_{i},g_{i}\right).$$

As for the 3D loss $L_{3D}$, a typical L1 loss with a heatmap regularization term is used to maximize the value at the 3D ground truth landmarks, ensuring the existence of a peak for each anatomical landmark:

(7) $$L_{3D}=\sum_{l}\left|y^{l}-y_{\mathrm{gt}}^{l}\right|-\alpha \cdot \log\left(V_{\mathrm{output}\;}^{l}\left(y_{\mathrm{gt}}^{l}\right)\right),$$

where α is a parameter weighting the influence of the second term.

## 3. Experiments and Results

### 3.1. Datasets

Comprehensive experiments were conducted on two typical yet challenging datasets of digitally reconstructed radiograph (DRR) images which were simulated from Computed Tomography (CT) scans. In our simulation system, the source-detector distance was set to 2000 mm, with an isocenter distance of 1000 mm. The projection was parameterized by the left/right anterior oblique (LAO/RAO) angle, which was randomly varied between $-15^{\circ }$ and $+15^{\circ }$, around the perfect AP and the LAT views. The 3D landmark ground truth was established by localizing vertebral body centers in the CT scans and then transforming the coordinates of the centers into the world coordinates system. The 2D landmark ground truth was obtained by projecting the 3D landmark ground truth to 2D image space.

**Lumbar Spine dataset**: The Lumbar Spine dataset contains DRR images generated from an in-house dataset of 130 CT scans, each containing the L1–L5 vertebrae. These CT scans were divided into three subsets: 100 for training, 10 for validation, and 20 for testing. For each of these CT scans, we generated 10 AP and 10 LAT views with a size of 1536×1024 pixels on a 450 mm × 300 mm detector plane, resulting in a total of 130×10×10 pairs of biplanar DRR images.

**Thoracic Spine dataset**: The Thoracic Spine dataset was generated from CT data collected from MICCAI (Medical Image Computing and Computer Assisted Interventions) VerSe19 and VerSe20 challenges [30]. A total of 235 CT scans containing thoracic vertebrae were selected and cropped into 1465 smaller volumes, each containing four consecutive thoracic vertebrae. These volumes were divided into three subsets: 993 for training, 153 for validation, and 319 for testing, ensuring that all volumes from the same scan were grouped in the same subset. For each CT scan, an AP and a LAT view were generated with a size of 1024×1024 pixels on a 300 mm × 300 mm detector plane.

### 3.2. Metrics

Three commonly used metrics are adopted to evaluate localization results.

**Point-to-point error (PE)**: The PE for each anatomical landmark is calculated as the Euclidean distance between the predicted and the ground truth landmark position. The mean and standard deviation of PE across all test images are reported, denoted as ${\mathrm{PE}}_{\mathrm{all}}$.

**Image-specific point-to-point error (IPE)**: The IPE for a specific image is the average of the PE values for that image. To provide a comprehensive overview, cumulative IPE distribution graphs, which can illustrate the proportion of images that reach various IPE values in our test dataset, are presented.

**Landmark identification rate (${\mathrm{ID}}_{\mathrm{rate}}$)**: The ${\mathrm{ID}}_{\mathrm{rate}}$ is the ratio between the accurately identified landmarks and the total number of vertebrae. A landmark is considered accurately identified if the distance between the predicted and the ground truth locations is below 5.0 mm.

### 3.3. Implementation Details

The proposed method was implemented with the PyTorch framework. The input images were rescaled to a size of 768×512 pixels for the Lumbar Spine dataset and a size of 512×512 pixels for the Thoracic Spine dataset. Empirically, the number of channels K for the 2D features *M* was set to 16, and the parameter α in Equation (7) was set to 0.01. The number of landmarks, *L*, that ProVLNet could detect was set to 5 for the Lumbar Spine dataset and 4 for the Thoracic Spine dataset. ProVLNet was trained for 1500 epochs on the Lumbar Spine dataset and 500 epochs on the Thoracic Spine dataset, considering the larger size of the Thoracic Spine dataset compared to the Lumbar Spine dataset. The Adam optimizer was adopted with a learning rate of 0.001 and a batch size of 4. All experiments were conducted on a single NVIDIA GeForce RTX 3090 GPU. To compare ProVLNet with other state-of-the-art (SOTA) methods, a Wilcoxon signed-rank test was performed with a significance level of 0.01.

### 3.4. Comparison Methods

In this study, our proposed ProVLNet was compared with two coordinate-level fusion methods [13,28] and three feature-level fusion methods [26,27]:

- 2D ResNet [28]: this method predicts 2D coordinates by a network based on ResNet-152 and determines the 3D coordinates by triangulation [26].
- 2D SCN [13]: this method predicts 2D coordinates by 2D SCN architecture [13] and determines the 3D coordinates by triangulation [26].
- Alg [26]: this is a baseline method introduced in [26], which enables gradient propagation for triangulating coordinates.
- Vol [26]: this is another method introduced in [26], which incorporates 3D information by unprojecting 2D features into 3D space.
- Adafuse [27]: this method fuses predicted 2D heatmaps based on epipolar geometry.

### 3.5. Results

#### 3.5.1. Results on the Lumbar Spine Dataset

**Figure 4:**
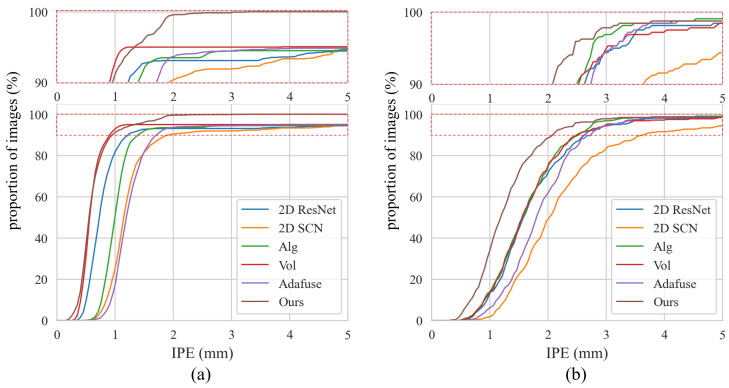
Cumulative distributions of IPE on the Lumbar Spine dataset (**a**) and the Thoracic Spine dataset (**b**). The top part shows a zoomed-in view of the dashed red box.

**Figure 5:**
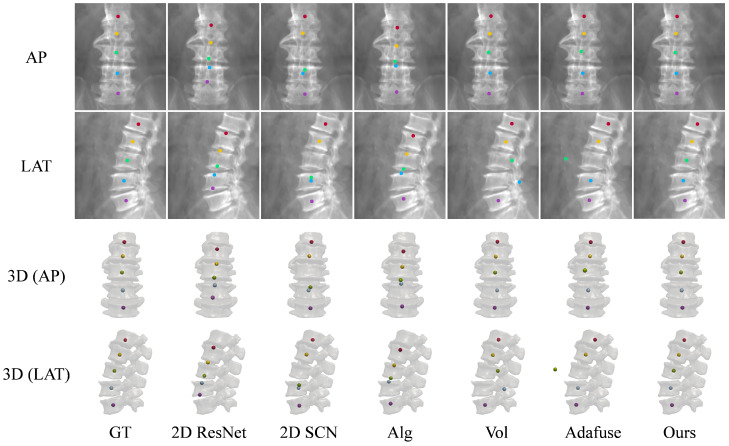
Landmark detection on the Lumbar Spine dataset using different methods: 2D ResNet [28], 2D SCN [13], Alg [26], Vol [26], and Adafuse [27]. The first two rows show the AP and the LAT views of projected landmarks, while the last two rows visualize landmarks in 3D space. The colored dots are the detected body centers of different lumbar vertebrae: red (L1), yellow (L2), green (L3), blue (L4), and purple (L5). GT: ground truth.

Table 2 compares the vertebra localization performance of ProVLNet with others on the Lumbar Spine dataset. ProVLNet outperforms other methods with an ${\mathrm{ID}}_{\mathrm{rate}}$ of 99.53% and a ${\mathrm{PE}}_{\mathrm{all}}$ of 0.64 mm. The ${\mathrm{PE}}_{\mathrm{all}}$ is reduced by about 50% over the second-best method (Vol [26]). Such improvement is statistically significant, as evidenced by the results of the Wilcoxon signed-rank test ($p=2.6\times 10^{-10}$). Figure 4a illustrates the cumulative IPE distributions for the Lumbar Spine dataset. The IPE of over 99% images provided by ProVLNet is under 3 mm, while the proportion of all other methods is below 95%. Figure 5 visualizes the localization results. ProVLNet identifies all vertebrae successfully, outperforming others.

#### 3.5.2. Results on the Thoracic Spine Dataset

**Figure 6:**
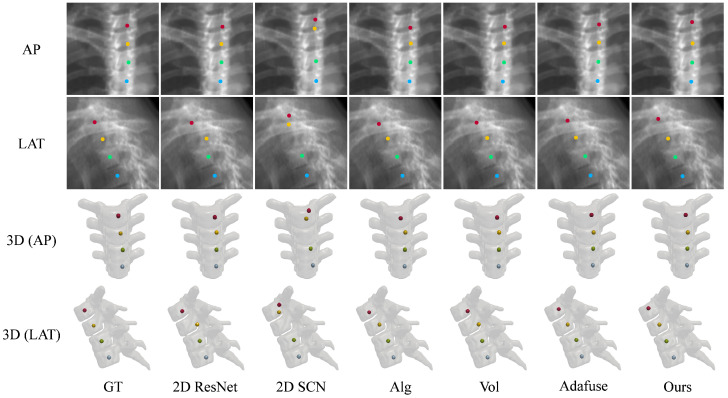
Landmark detection on the Thoracic Spine dataset using different methods: 2D ResNet [28], 2D SCN [13], Alg [26], Vol [26], and Adafuse [27]. The first two rows show the AP and the LAT views of projected landmarks, while the last two rows visualize landmarks in 3D space. The colored dots are the detected body centers of different thoracic vertebrae. GT: ground truth.

Table 3 compares the vertebra localization performance of ProVLNet with others on the Thoracic Spine dataset. As one can see from this table, in comparison with other SOTA methods, ProVLNet achieves the best results with an ${\mathrm{ID}}_{\mathrm{rate}}$ of 98.98% and a ${\mathrm{PE}}_{\mathrm{all}}$ of 1.38 mm. The ${\mathrm{PE}}_{\mathrm{all}}$ is reduced by about 20% over the second-best method (Alg [26]) ($p=1.1\times 10^{-9}$). Figure 4b illustrates the cumulative IPE distributions for the Thoracic Spine dataset. As one can see from this figure, ProVLNet prevails over other SOTA methods with the highest proportion of images in the range of 0.5–4 mm. The localization results are visualized in Figure 6. Again, ProVLNet performs localization with the best precision, particularly for the last two vertebrae in the lateral view.

#### 3.5.3. Ablation Study

**Figure 7:**
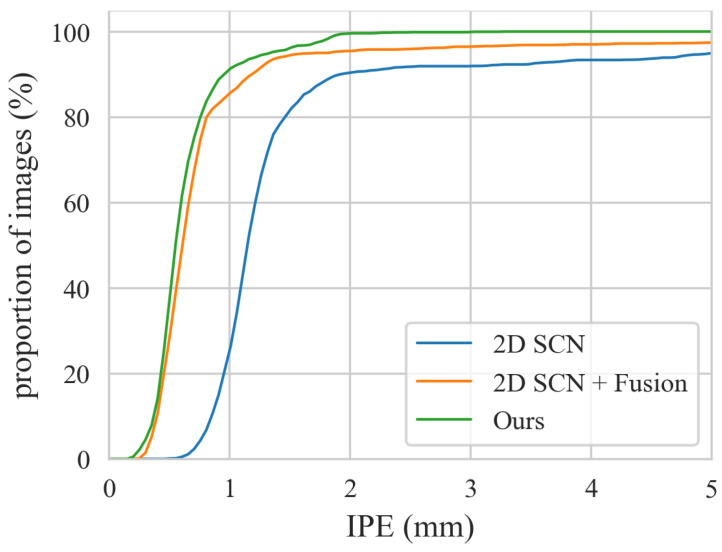
Cumulative distributions of IPE on the Lumbar Spine dataset of ablation study.

An ablation study on the Lumbar Spine dataset was conducted to evaluate the effectiveness of the spatial alignment fusion module and the 3D landmark regression module. The results are presented in Table 4. The baseline, represented in the first row, is the backbone network of the Siamese 2D feature extractor, consistent with the 2D SCN [13] in Table 2. The approach in the second row integrates the 2D SCN and the spatial alignment fusion module with the 3D Convolutional Neural Network (CNN) from the Vol method [26] to determine landmark coordinates. According to Table 4, incorporating the spatial alignment fusion module leads to a 0.73% increase in ${\mathrm{ID}}_{\mathrm{rate}}$, and the addition of the 3D landmark regression module contributes to a further 1.5% improvement. Correspondingly, the ${\mathrm{PE}}_{\mathrm{all}}$ metric shows improvements of 0.62 mm and 0.36 mm, respectively. Figure 7 shows the cumulative distributions of IPE for the methods in Table 4. It shows that for all IPE values, our method achieves the highest proportion.

#### 3.5.4. Analysis of Intermediate Features

**Figure 8:**
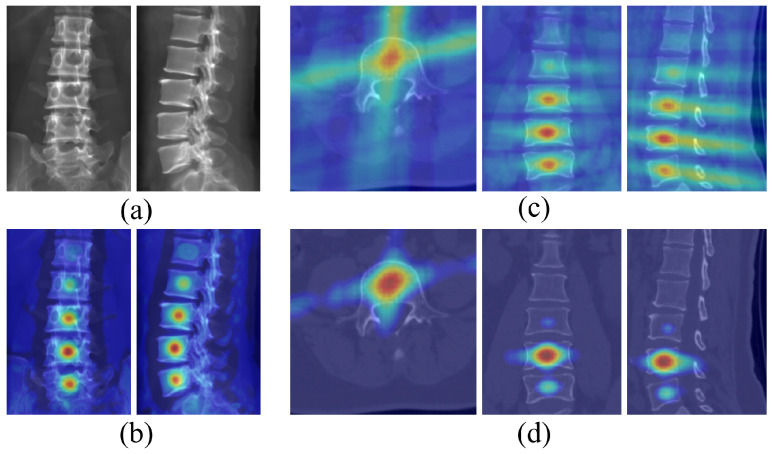
Visualization of the intermediate features when detecting the body center of the L4 vertebra from a given pair of AP and LAT images. (**a**) Original input image; (**b**) output from the Siamese 2D feature extractor; (**c**) 3D unprojection of features before 3D SCN (transverse, coronal, sagittal views); (**d**) output after 3D SCN (transverse, coronal, sagittal views).

Qualitatively, Figure 8 shows the intermediate features when detecting the body center of the L4 vertebra from a given pair of AP and LAT images, which can be used to illustrate the efficacy of ProVLNet. Specifically, using the images from Figure 8a as inputs, the Siamese 2D feature extractor generates aligned local appearance features, with one representative channel shown in Figure 8b. These features effectively highlight the detected vertebral body centers, but the ambiguity between adjacent vertebrae remains. The spatial alignment fusion module then fuses these features based on projective geometry and outputs the aggregated 3D features displayed in Figure 8c. The aggregated 3D features from the spatial alignment fusion module highlight areas at the 3D vertebral body centers, capturing the underlying 3D information. Following this, the 3D SCN, which is a component of the 3D landmark regression module, outputs the predicted heatmaps as shown in Figure 8d. Each channel in these heatmaps represents a specific landmark. As one can see from Figure 8d, the ambiguity is resolved by the 3D SCN, where false positive responses in adjacent vertebrae shown in Figure 8c are suppressed.

## 4. Discussion and Conclusions

In this paper, an end-to-end network referred to as ProVLNet was proposed. ProVLNet was designed to incorporate projective geometry for accurate localization of vertebrae in 3D space from calibrated biplanar X-ray images. In particular, 2D local appearance features were first extracted by a Siamese 2D feature extractor. The extracted 2D appearance features were then fused in 3D space by a carefully designed spatial alignment fusion module. Finally, 3D coordinates of all landmarks were predicted by a 3D landmark regression module. Comprehensive experiments were conducted on two typical yet challenging datasets to validate the efficacy of the proposed ProVLNet. Quantitatively and qualitatively, the experimental results demonstrated the superior performance of the proposed ProVLNet over other SOTA methods.

It is apparent that the coordinate-level fusion methods such as 2D ResNet [28] and the 2D SCN [13] generate suboptimal results, as demonstrated by the quantitative results presented in Table 2 and Table 3. This is largely due to the fact that these methods learn to detect 2D landmarks from the AP and the LAT image independently, followed by a 3D coordinate triangulation to generate the final results. Thus, rather than incorporating the projection geometry into the learning process, these methods only use it in the coordinate triangulation step, leading to suboptimal results. Although methods such as Alg [26] and Adafuse [27] exploit epipolar geometry to integrate biplanar information, they do not incorporate underlying 3D information, resulting in lower performance. In contrast, by incorporating the projection geometry into the learning process and by including a 3D landmark regression module, our proposed ProVLNet can not only implicitly model the 3D anatomical landmark prior but also reasonably handle ambiguity in landmark detections, leading to superior results on both datasets.

It is worth comparing the proposed ProVLNet with the Vol method [26] as both methods are designed to incorporate 3D information. Compared with the Vol method [26], our method benefited from a decomposition strategy that divided the main task into two sub-problems: the extractions of 2D features with ambiguous candidate predictions and the reduction in ambiguity in 3D space. Such a strategy was proved to be effective for anatomical landmark detection tasks [13], as demonstrated quantitatively and qualitatively by the results presented in Table 2 and Table 3 and Figure 4, Figure 5 and Figure 6.

The effectiveness of the carefully designed spatial alignment fusion module and the 3D landmark regression module was demonstrated by the ablation results shown in Table 4 and Figure 8. By unprojecting 2D features into 3D space based on projective geometry, the spatial alignment fusion module captured the underlying 3D information, as demonstrated by an example shown in Figure 8c. The 3D landmark regression module further resolved the ambiguity in landmark detections by suppressing false positive responses in adjacent vertebrae, as demonstrated by an example shown in Figure 8d.

There exist limitations in the present study. First, the number of vertebrae in the calibrated biplanar images that ProVLNet can detect was fixed, i.e., 5 for the Lumbar Spine dataset and 4 for the Thoracic Spine dataset. Extending our method to handle the arbitrary number of vertebrae will be our future work. Second, due to the difficulty in organizing calibrated biplanar X-ray images in clinical scenarios, we only validated our method on synthetic datasets. One way to generalize the trained models to calibrated biplanar X-ray images in clinical scenarios in the future is to explore unsupervised domain adaptation technique [31]. Nevertheless, since all the methods were compared on the same datasets, the results that we obtained in this study demonstrated the superior performance of ProVLNet over other SOTA methods.

In summary, we proposed a projective-geometry-aware network called ProVLNet to localize 3D vertebrae in calibrated biplanar X-ray images. It incorporates 3D information into the landmark detection process via a carefully designed spatial alignment fusion module. The remaining ambiguity in landmark detections are further resolved by the 3D landmark regression module. ProVLNet outperformed other SOTA methods when evaluated on two typical and challenging datasets acquired for the lumbar and the thoracic spine. It holds the potential to be applied to clinical scenarios of X-ray-guided spine surgery.

## Figures and Tables

**Table 1:** Overview of the state-of-the-art (SOTA) dual-view localization methods.

Methods	Backbone	Fusion Level	Fusion Strategy
2D ResNet [28]	ResNet	Coordinate-level	Triangulation of 2D coordinates
2D SCN [13]	SCN	Coordinate-level	Triangulation of 2D coordinates
Alg [26]	ResNet	Feature-level	Gradient-based triangulation
Vol [26]	ResNet	Feature-level	Unprojecting 2D features into 3D space
Adafuse [27]	ResNet	Feature-level	Fusion of predicted heatmaps in 3D space
Ours	SCN	Feature-level	Unprojecting 2D features into 3D space

**Table 2:** Overview of vertebra localization performance comparison on the Lumbar Spine dataset (mean ± SD). The best results are highlighted in bold.

Method	${\mathrm{ID}}_{\mathrm{rate}}$ (%)	${\mathrm{PE}}_{\mathrm{all}}$ (mm)
2D ResNet [28]	95.68	1.53 ± 3.95
2D SCN [13]	97.30	1.62 ± 3.08
Alg [26]	96.18	1.72 ± 3.88
Vol [26]	97.76	1.27 ± 5.42
Adafuse [27]	96.97	3.98 ± 19.02
Ours	**99.53**	**0.64 ± 0.57**

**Table 3:** Overview of vertebra localization performance comparison on the Thoracic Spine dataset (mean ± SD). The best results are highlighted in bold.

Method	${\mathrm{ID}}_{\mathrm{rate}}$ (%)	${\mathrm{PE}}_{\mathrm{all}}$ (mm)
2D ResNet [28]	98.59	1.76 ± 1.23
2D SCN [13]	94.67	2.40 ± 2.17
Alg [26]	98.74	1.68 ± 1.08
Vol [26]	98.43	1.73 ± 1.25
Adafuse [27]	98.74	1.92 ± 1.08
Ours	**98.98**	**1.38 ± 1.72**

**Table 4:** Quantitative results of the ablation study on the Lumbar Spine dataset. The best results are highlighted in bold. Fusion: spatial alignment fusion.

	Components			Results	
Method	2D SCN	Fusion	3D Landmark Regression	${\mathrm{ID}}_{\mathrm{rate}}$ (%)	${\mathrm{PE}}_{\mathrm{all}}$ (mm)
2D SCN	✓			97.30	1.62 ± 3.08
2D SCN + Fusion	✓	✓		98.03	1.00 ± 2.56
Ours	✓	✓	✓	**99.53**	**0.64 ± 0.57**

## Data Availability

The data presented in this study are available on reasonable request from the corresponding author.

## Abbreviations

The following abbreviations are used in this manuscript:

AP	Anterior–posterior
CT	Computed Tomography
DRR	Digitally reconstructed radiograph
LAO	Left anterior oblique
LAT	Lateral
MICCAI	Medical Image Computing and Computer Assisted Interventions
RAO	Right anterior oblique
SCN	SpatialConfiguration-Net
SOTA	State-of-the-art
2D	Two-dimensional
3D	Three-dimensional
